# Development and implementation of a strategy for intensified screening for *gambiense* human African trypanosomiasis in Kongo Central province, DRC

**DOI:** 10.1371/journal.pntd.0008779

**Published:** 2020-10-15

**Authors:** Crispin Lumbala, Simon Kayembe, Jacquies Makabuza, Pascal Lutumba, Jean-Pierre Van Geertruyden, Paul R. Bessell, Joseph Mathu Ndung’u

**Affiliations:** 1 Directorate of Disease Control, Ministry of Public Health, Democratic Republic of the Congo; 2 Global Health Institute, Faculty of Medicine and Health Sciences, University of Antwerp, Antwerp, Belgium; 3 Foundation for Innovative New Diagnostics (FIND), Geneva, Switzerland; 4 Programme National de Lutte Contre la Trypanosomiase Humaine Africaine, Kinshasa, République Démocratique du Congo; 5 Kinshasa University, Kinshasa, Democratic Republic of the Congo; 6 Epi Interventions, Edinburgh, United Kingdom; Universidade do Estado do Rio de Janeiro, BRAZIL

## Abstract

**Background:**

The Democratic Republic of the Congo (DRC) accounts for the majority of the reported *gambiense* human African trypanosomiasis (HAT) cases. Kongo Central province in the DRC reports a relatively low, yet steady number of cases, and forms a transboundary focus with Angola and the Republic of Congo. This paper describes an intervention aimed at reducing the case burden in Kongo Central by improving passive case detection, complemented with reactive screening.

**Methodology/Principal findings:**

At the initiation of this programme in August 2015, 620 health facilities were identified and equipped with Rapid Diagnostic Tests (RDTs) for HAT screening. Of these, 603 (97%) reported use of RDTs, and 584 (94%) that continued to use RDTs to the last quarter of 2016 were used in the analysis going forward. Among all health facilities involved, 23 were equipped to confirm HAT by microscopy, and 4 of the latter were equipped to perform molecular testing with loop-mediated isothermal amplification (LAMP). Patients clinically suspected of HAT were tested with an RDT and those with a positive RDT result were referred to the nearest microscopy facility for confirmatory testing. If RDT positive patients were negative by microscopy, they were tested by LAMP, either on fresh blood or blood that was dried on filter paper and transported to a facility performing LAMP. This network of diagnostic facilities reduced the median distance for a patient to travel to a screening facility from 13.7km when the classical card agglutination test for trypanosomiasis (CATT) was used as a screening test in the past, to 3.4km. As a consequence, passive case detection was improved by between 30% and 130% compared to the period before. Furthermore, the proportion of HAT cases detected in early stage disease by passive screening increased from 27% to 64%.

Reactive screening took place in 20 villages where cases were reported by passive screening, and in 45 villages in the neighbourhood of these villages. Reactive screening was responsible for detection of 40% of cases, of which, 90% were in first stage of the disease.

**Conclusions:**

This programme has demonstrated that it is possible to deploy passive screening for HAT at sub-country or country levels in the DRC, and this is made more effective when supplemented with reactive screening. Results and achievements showed an increase in the number of HAT cases detected, the majority of them in early disease, demonstrating that this strategy enables better population coverage and early detection of cases, which is critical in removing the HAT reservoir and interrupting transmission, and could contribute to HAT elimination in regions where it is implemented.

## Author summary

A number of diagnostic tests for HAT have recently been developed, to improve case detection. We report on the use of these technologies in a strategy to increase coverage and early detection of HAT cases in Kongo Central province of DRC.

All 620 health facilities in the focus were equipped with RDTs to test patients presenting with symptoms suggestive of HAT. Among these health facilities, 23 were upgraded to perform confirmatory testing, for a final diagnosis.

This strategy has reduced the distance a patient travels to a facility screening for HAT, from 13.7km to 3.4km. From August 2015 to December 2016, the proportion of HAT cases detected, adjusted annually, increased by between 30% and 130% compared to the previous two years, and 64% of them were in early stage disease, compared to 27% previously. This strategy has enabled better population coverage, and when supplemented with reactive screening, the identification of local outbreaks and early detection of most cases, which is critical in removing the HAT reservoir and interrupting transmission, thus contributing to elimination of the disease.

## Introduction

Human African trypanosomiasis (HAT), also known as sleeping sickness, is a parasitic disease transmitted by the bite of an infected tsetse fly (*Glossina spp*). The disease is endemic in sub-Saharan Africa, within the limits of the geographic distribution of the tsetse fly. The disease is caused by protozoan parasites belonging to the species *Trypanosoma brucei (T*.*b*.*)*. The majority of cases of HAT are caused by the sub-species *T*.*b*. *gambiense*, which accounts for all cases reported in West and Central Africa [1]. In many HAT endemic areas, no vector control is carried out, and control of HAT relies on the detection and treatment of infected individuals. This is made difficult by the requirement for case confirmation through visualisation of the parasites in body fluids by microscopy, which in itself has imperfect sensitivity.

In recent years, a number of new technologies have been developed that overcome many of these challenges, including rapid diagnostic tests (RDTs) [2–5] and Light emitting diode (LED) fluorescence microscopy (FM) [6, 7], and in part, the imperfect sensitivity of confirmatory testing has been improved by loop-mediated isothermal amplification (LAMP) of DNA [8, 9]. RDTs do not require electricity to perform, and they can be deployed in any health facility where staff are familiar with use of RDTs, while LED FM has low requirements for electricity, and can be powered by solar panels. These new technologies, in combination with other existing tools, has enabled implementation of a new diagnostic algorithm for HAT, in which clinically suspected patients are first screened with RDTs, and if positive, or if they have clinical signs strongly suggestive of HAT, they are referred to a facility with the capacity for confirmatory testing. Those that are negative with the confirmatory test are subjected to LAMP on fresh blood in sites upgraded to perform LAMP, or on blood samples dried on filter paper collected in confirmatory sites that are not equipped to perform LAMP. At the commencement of the project in August 2015, this algorithm had been implemented successfully in Uganda for two years [10].

**Fig 1:**
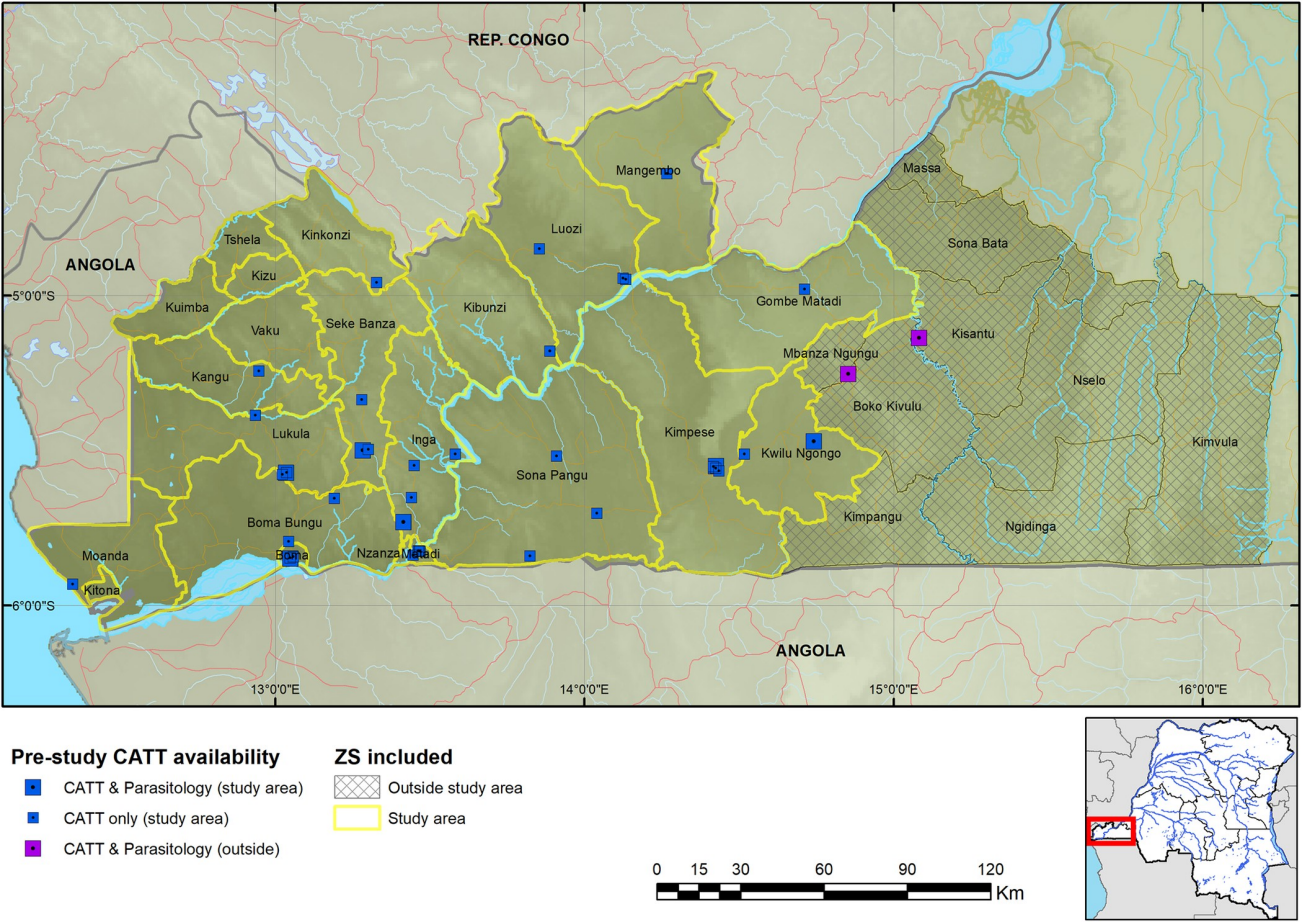
Map of sites performing CATT in Kongo Central province during 2013 and 2014. The geodata layers were obtained from CC-BY License compatible sources: The CGIAR SRTM 90m datasets (https://bigdata.cgiar.org/srtm-90m-digital-elevation-database/). Further geodata were downloaded from Digital Chart of the World through Diva-GIS (https://www.diva-gis.org/gdata) and OpenStreetMaps (http://www.openstreetmap.org) and rendered by the authors using ESRI ArcGIS following the PLOS guidelines (https://journals.plos.org/plosone/s/figures) or were supplied by PNLTHA.

While the Democratic Republic of the Congo (DRC) has always reported the greatest case burden annually, prior to, and since initiation of this programme, the number of cases reported in the country has been falling [11]. Active screening by mobile teams moving to HAT endemic villages has until recently been the main strategy to control HAT in DRC. From 2000 to 2012, an average of 90% (ranging from 72% to 95%) of the population in endemic areas were actively screened. The proportion of HAT cases detected by active screening was almost equal to the one detected through passive screening in health facilities, averaging 53%, and a range of 47% to 63%. However, majority of the cases have been diagnosed in early stage through active screening by mobile teams, with the proportion ranging from 62% to 83% and averaging 76%. This could be explained by different challenges faced by passive case detection, such as the fact that people present to health facilities after having persistent signs and symptoms of HAT and/or after visiting different health facilities over lengthy periods of time. In addition, the tests used for screening required electricity and a cold chain, limiting expansion of the diagnostic capacity to available health facilities. [11–14]. Different challenges associated with passive case detection limited passive screening in Kongo Central province to 40 facilities within the study area (of which seven could perform the capillary tube centrifugation technique—CTC and/or the mini-anion exchange centrifugation technique—mAECT, the most sensitive confirmatory parasitological tests for HAT) and two smaller facilities that were outside the study area, where no HAT case was diagnosed for over 5 years prior to the intervention, since 2013 (Fig 1 & S1 Data).

Among the HAT endemic regions in the DRC, the focus in Kongo Central province, formerly Bas Congo, is a transboundary focus bordering endemic areas in the Republic of Congo to the north and Angola to the south. Furthermore, the number of cases reported in Kongo Central in the years before the project had been low–typically between 100 and 200 cases per year, which is relatively few in the context of DRC (Table 1). The reported low number of cases in this HAT focus indicates that a sustained drive to diagnose cases could lead to local elimination of the disease in the region. Furthermore, the province is also one of the smaller ones in the DRC.

**Table 1:** Number of HAT cases reported in various provinces (historical) in the DRC from 2010 to 2015.

Former province	Number of HAT cases					
	2010	2011	2012	2013	2014	2015
Bas Congo (Kongo Central)	167	105	107	130	70	83
Bandundu	2,925	2,506	3,167	2,415	1,885	1,575
Equateur	308	321	197	71	75	47
Kasai Occidental	270	356	171	132	98	118
Kasai Oriental	697	698	701	502	313	266
Katanga	52	62	91	133	64	49
Kinshasa	153	143	134	166	103	83
Maniema	71	53	128	104	63	57
Province Orientale	986	1,351	1,282	1,971	535	71
Other	0	0	0	0	0	0
Total	5,629	5,595	5,978	5,624	3,206	2,349

Data from Lumbala et al, 2015 [11].

In this paper, we report on a programme to improve the coverage of HAT case detection in Kongo Central through an expansion of passive screening to include all health facilities in the Kongo Central HAT focus, complemented by targeted reactive screening in sampled villages that report cases. Success was measured by the number of HAT cases reported, where they were screened, and the disease stage those cases were in. The programme was initiated in August 2015 and implemented in all health facilities in the selected region by November 2015.

## Methods

The core phases of the study involved the development of a diagnostic algorithm, identification and characterisation of health facilities in the province, the selection of those that were suitable for upgrading to perform parasitological techniques and those that were suitable for LAMP. The strategies for monitoring activities and identifying villages in which reactive screening was to be performed were also established.

### Health facility characterization

Characterization of health facilities was a process of gathering as much information about the facility using a questionnaire, and its location using a hand-held GPS instrument (S1 Text). The questionnaire, which was completed by the health facility manager, was brief and comprised of questions on the current staffing and laboratory capacity of the facility, as well as the history of diagnosing HAT. A member of the national HAT control programme visited each health zone and trained local extension nurses in facility characterization and use of the GPS. The nurses would then visit each facility and carry out the characterization. The data would subsequently undergo quality control and any adjustments made. These data were then mapped (Fig 1).

### Upgrading of health facilities

The health facility characterization data were analysed to optimally deploy resources for upgrading facilities to perform microscopy and LAMP. Based on the information gathered, 19 facilities were upgraded to perform microscopy (LED FM sites) and 4 to perform microscopy and LAMP (LAMP sites). The following criteria were considered in identifying the facilities to be upgraded:

- The current HAT diagnostic capacity and the history of diagnosing HAT.
- The laboratory and human resource capacity. Microscopy requires a lab with trained staff, and electricity can be provided by installation of solar panels. LAMP requires highly trained staff and a reliable source of electricity.
- The numbers of HAT cases reported in the locality.

All staff involved in the programme received training in clinical diagnosis of HAT, use of the tests, and the procedures for referral, depending on activities implemented in the facility.

### Diagnostic algorithm

The diagnostic algorithm for passive screening adopted in Kongo Central was similar to the one described in Wamboga et al., (2017), and is shown in S1 Fig “The *T*. *b*. *gambiense* human African trypanosomiasis (gHAT) diagnostic workflow implemented in Kongo Central”. Briefly, patients presenting themselves at health facilities with clinical signs indicative of HAT, who test negative with malaria RDTs, since malaria presents similar signs and symptoms, are screened using a HAT RDT, the second level screening for HAT following clinical screening based on symptomatology. Those that are positive with the HAT RDT are tested by microscopy in an algorithm comprising palpation and puncture of lymph nodes (LN) if enlarged, and examination of the aspirate, LED FM on thick smears, CTC, LED FM on thin smears of lysed blood, and mAECT on buffy coat (mAECT-bc). If patients test positive with the HAT RDT at facilities that perform only HAT RDTs (RDT sites) they are referred to facilities equipped to perform microscopy (microscopy or LED FM site). If positive with a confirmatory test, the patient’s stage of disease is determined and treatment carried out according to national guidelines. If negative, samples of whole blood and buffy coat are dried onto a filter paper and transported using a programme motorcycle to the nearest LAMP facility (LAMP site) for testing. If a patient is HAT RDT positive and microscopy negative at a facility with the capacity to perform LAMP, this test is performed on fresh samples. If either sample is positive, then the patient is recalled for further testing by microscopy, and if negative, no further testing is undertaken, but the patient is asked to present in three months for repeat testing with an RDT.

The RDTs used in this programme were the SD BIOLINE Malaria Antigen *Plasmodium falciparum* test (SD Malaria *P*.*f*. RDT) for malaria diagnosis and the SD BIOLINE HAT RDT (HAT RDT) for HAT screening. Both are cassette-format RDTs developed by Standard Diagnostics, Inc. (SD, Geonggi-do, South-Korea), now Abbott Diagnostics, Korea Inc–ADK, Republic of Korea.

The Malaria *P*.*f*. RDT includes one test band for the qualitative detection of the *P*. *falciparum* Histidine Rich Protein II (HRP-II or HRP2) antigen (Ag) [15] in human whole blood. The SD BIOLINE HAT RDT includes two test bands to detect antibodies against two trypanosome variable surface glycoprotein (VSG) antigens (LiTat 1.3 and LiTat 1.5) [2, 3].

During reactive screening, patients were screened using the card agglutination test for trypanosomiasis (CATT) or CATT and RDTs in parallel, and if either was positive, then the patient underwent a series of microscopy tests in the following order: LN-CTC-mAECT. Patients found positive using this algorithm were staged and referred for treatment. Suspects that were negative had samples of whole blood and buffy coat dried on filter paper and transported to a facility performing LAMP. Those positive by LAMP were recalled for further testing by microscopy.

### Monitoring of activities

Activities were monitored through a combination of monthly reporting by all RDT facilities by SMS, phone call or email to the local coordinator, on the number of patients tested and numbers positive. Facilities performing microscopy and LAMP also submitted details on tests performed, their results, disease stage and village of origin of any cases. These data were analysed and mapped as they came in.

### Reactive screening

In villages from which cases were identified by passive screening, a follow up reactive screening was scheduled. At a date in the following months (when logistically possible) a mobile team using either a vehicle or motorbikes (mini-mobile team) visited a number of sampled and neighbouring villages and screened all persons that presented themselves. This followed the national guidelines [16] but used the diagnostic algorithm described above, and was done using either CATT or both CATT and RDTs in parallel.

### Metrics of activities’ results

To drive elimination of HAT, it is necessary to maximise the number of people screened among the at-risk population, in efforts to identify most cases early during the course of infection [17, 18]. Thus, success of the programme is primarily measured through comparison of the number of cases identified by passive screening before, versus during the programme, and by analysis of the proportions of these cases that were in the early stages of HAT. Further metrics include the coverage, the HAT RDTs usage at different facility levels, and the proportions of serological suspects from RDT facilities that were successfully referred for confirmation. The coverage was evaluated in terms of distance to the health facility, and of the population within a 5-8kms radius around sites. This was evaluated by using data extracted from the WorldPop gridded population data sets and overlaying the raster layer of the distances to health facilities [19]. According to DRC health policies, a health facility should cover a population in a radius of 5-8kms [20]. The coverage is the percentage of the population within 5-8kms radius of each of the health facilities that implemented the project and screened patients using HAT RDTs, over the total population in the selected region.

### Ethical considerations

This project was carried out in conformity with the Declaration of Helsinki, and was approved by the Ethics Committee of the School of Public Health, University of Kinshasa, DRC (Letter Ref. No ESP/CE/049/14, October 02^nd^, 2014). Clinical use of HAT RDTs in DRC was approved in 2014 (Letter Ref. No. MS.1251/SG/THA/774/MK/2014, from the General Secretary, Ministry of Public Health). Management of HAT cases was done in accordance with DRC national guidelines; only patients in whom trypanosomes were visualized by microscopy in body fluids were treated as HAT cases [16].

## Results

### Roll out of activities

**Fig 2:**
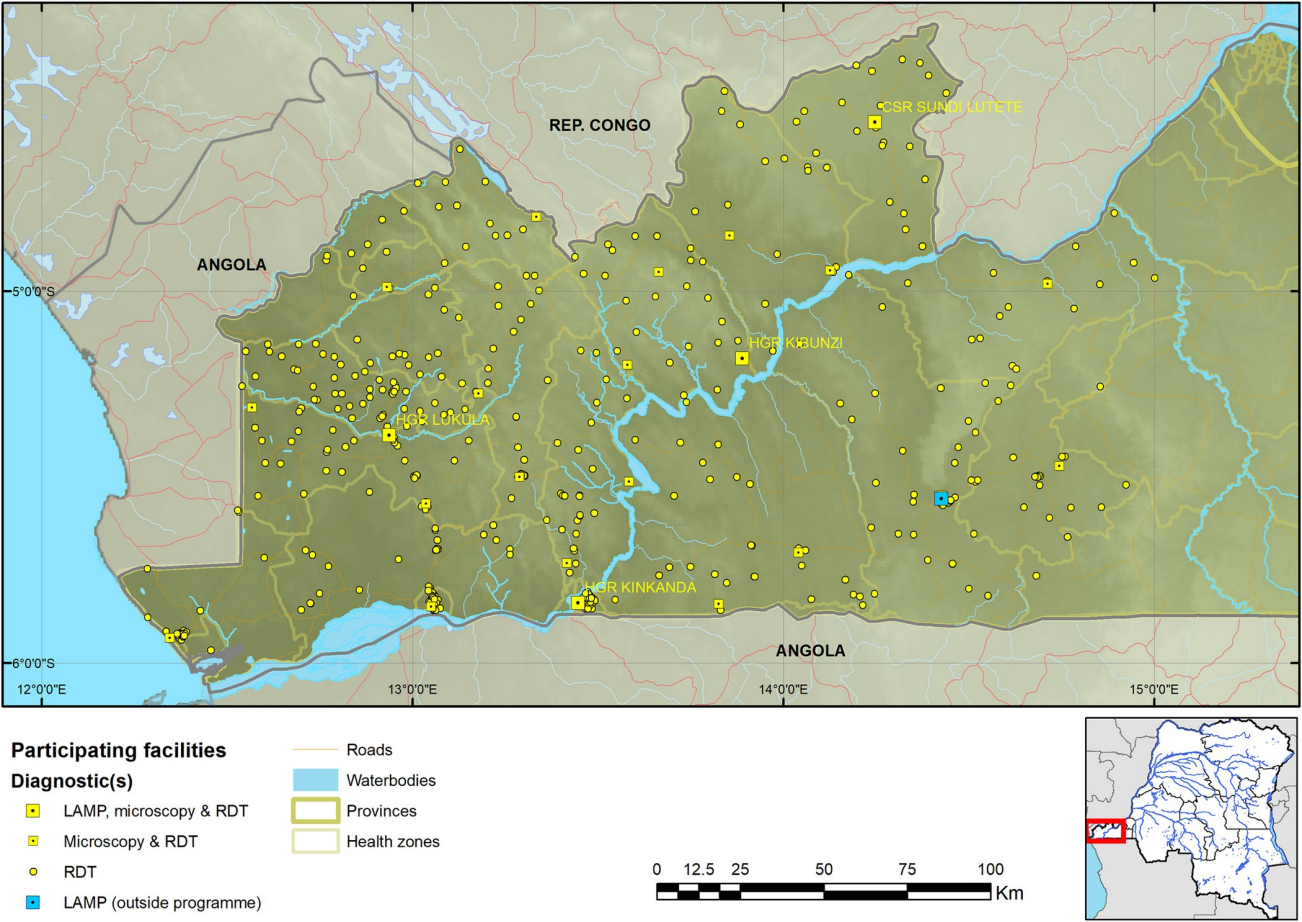
Map of RDT, LED FM and LAMP sites in the Kongo Central province programme. The geodata layers were obtained from CC-BY License compatible sources: The CGIAR SRTM 90m datasets (https://bigdata.cgiar.org/srtm-90m-digital-elevation-database/). Further geodata were downloaded from Digital Chart of the World through Diva-GIS (https://www.diva-gis.org/gdata) and OpenStreetMaps (http://www.openstreetmap.org) and rendered by the authors using ESRI ArcGIS following the PLOS guidelines (https://journals.plos.org/plosone/s/figures) or were supplied by PNLTHA.

Kongo Central province has 31 health zones, of which Massa, situated in the north eastern region of the province, is at the edge of the epidemiological extent of the HAT focus in the province. Among the other 30 health zones, 17 reported at least one HAT case from 2009 to 2013 (and are hereafter referred to as the HAT endemic health zones). RDTs were deployed in 620 facilities across 19 health zones (S1 Data), including the 17 HAT endemic health zones, and Nzanza and Vaku health zones, which had not reported cases from 2009 to 2013, but are surrounded by HAT endemic health zones. Among the 620 health facilities, 23 were equipped to perform microscopy, and among these 23 health facilities, 4 were upgraded to perform LAMP. Of these 620 facilities, 603 started using RDTs and by the final quarter of 2016, 584 were still using RDTs. These 584 facilities tested a total of 45,173 patients with HAT RDTs, and it is these 584 that were used in the analysis going forward (374 patients were screened by the 19 facilities that ceased using RDTs by the final quarter of 2016 and reporting, no cases were confirmed among these). This brought to 561 the number of RDT sites (performing only HAT RDTs), 19 LED FM sites (performing HAT RDT and microscopy) and 4 LAMP sites (performing HAT RDT, microscopy and LAMP). Enrolment of facilities was carried out gradually, starting in August 2015, with the final facilities enrolled in November 2015. All facilities except 41 RDT sites were mapped (Figs 1 & 2); for the latter, GPS coordinates were not collected by provincial and health zone teams.

### Coverage

**Fig 3:**
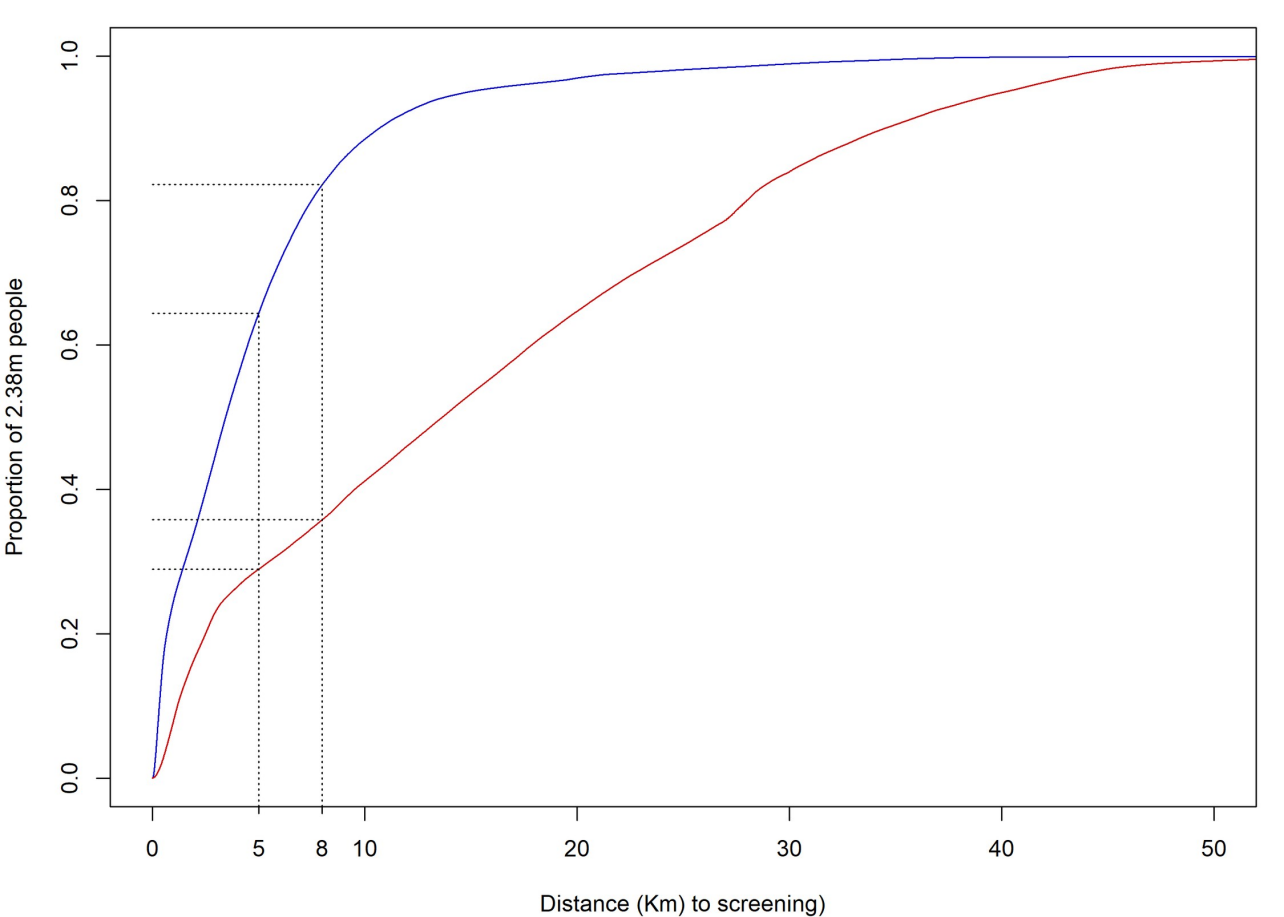
Cumulative distribution plot of the population’s distances to screening facilities using HAT RDTs or CATT. The red line represents the distance to HAT screening prior to the programme, the blue line is the distance following the start of the programme and the black lines the coverage at the 5 and 8km targets of the DRC Ministry of Health. Data on population are from [19].

**Fig 4:**
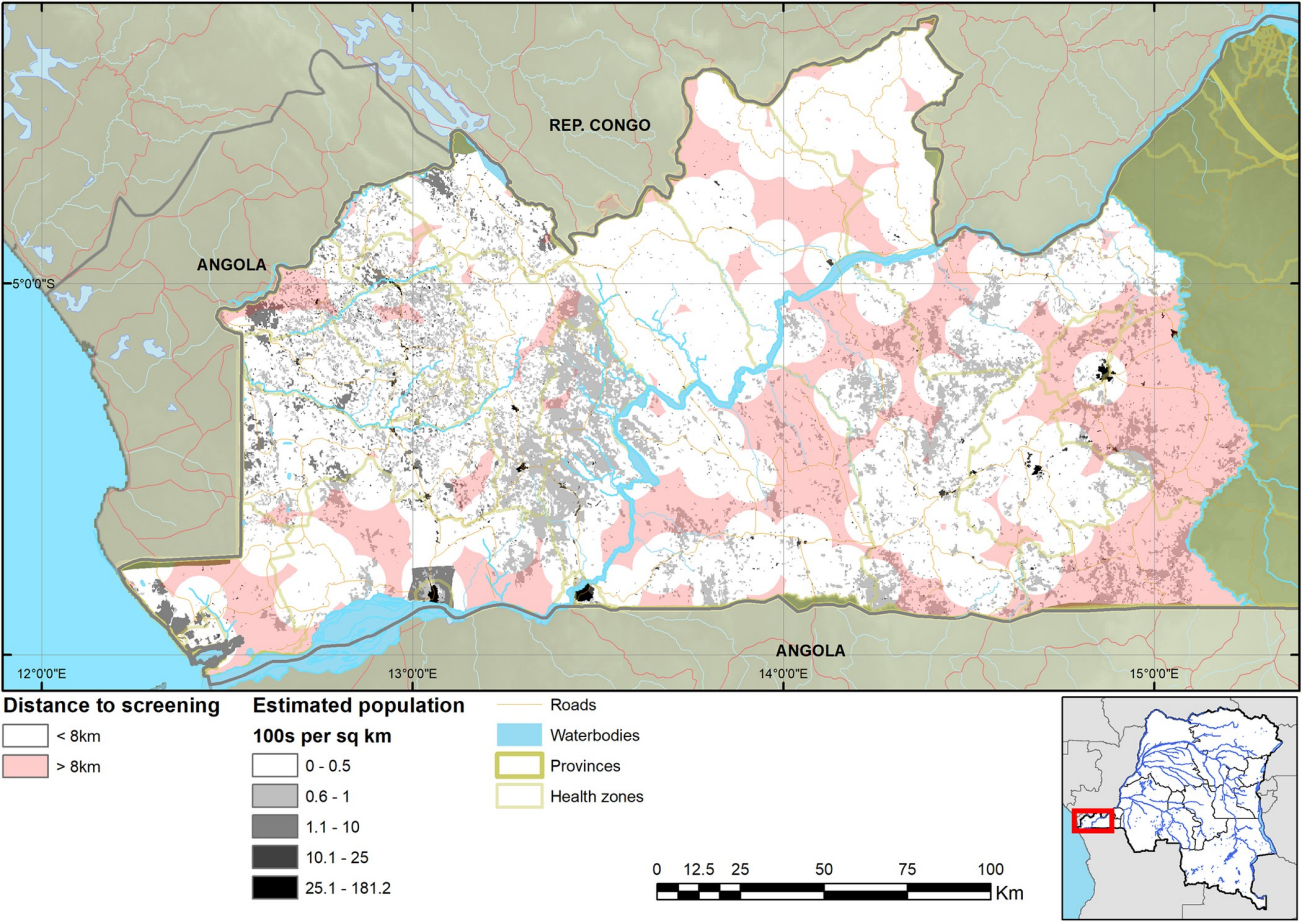
Map of estimated population within and beyond 8km distance to health facilities screening for HAT. The geodata layers were obtained from CC-BY License compatible sources: The CGIAR SRTM 90m datasets (https://bigdata.cgiar.org/srtm-90m-digital-elevation-database/). Further geodata were downloaded from Digital Chart of the World through Diva-GIS (https://www.diva-gis.org/gdata) and OpenStreetMaps (http://www.openstreetmap.org) and rendered by the authors using ESRI ArcGIS following the PLOS guidelines (https://journals.plos.org/plosone/s/figures) or were supplied by PNLTHA. Data on population are from [19].

As a result of the expansion of HAT screening activities, the median straight-line distance of the population to a facility that performed HAT RDTs is 3.4km, with 95% within 14.7km from a facility (4.9km mean distance). Comparing this to the situation when CATT was the only screening test, the median was 13.7km, with 95% of the population within 40.0km (Fig 2). The median distance for referral to microscopy is 12.0km and the corresponding mean distance 12.4km. For screening with RDTs, 64.4% of the population lives less than 5km and 82.2% less than 8km from an RDT facility, compared to screening with CATT in the past when only 28.9% lived within 5km and 35.8% within 8km from a CATT facility (Figs 3 and 4).

### RDT usage

**Fig 5:**
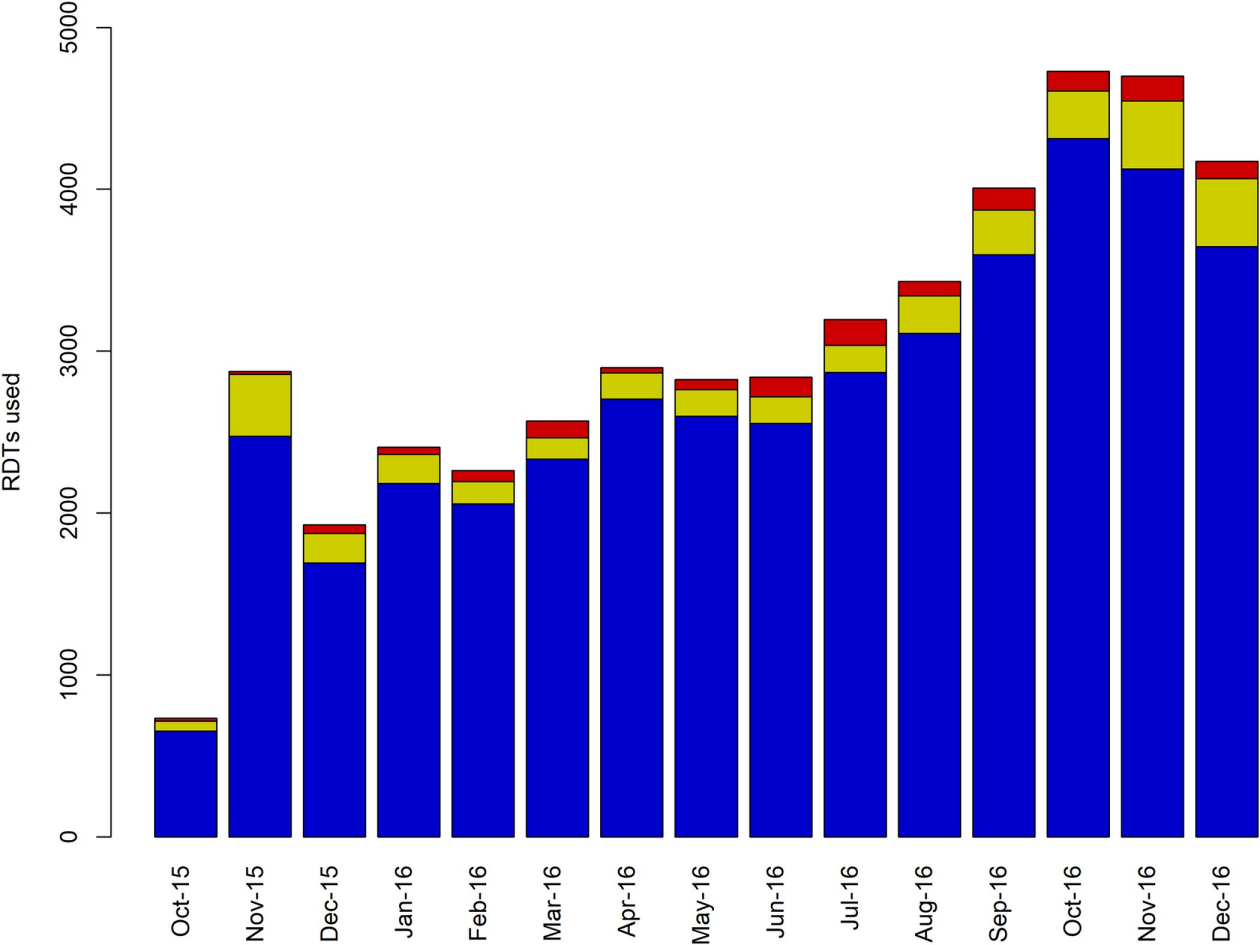
Number of patients screened with HAT RDTs per month. Blue represents RDT facilities, yellow microscopy facilities and red LAMP facilities.

**Fig 6:**
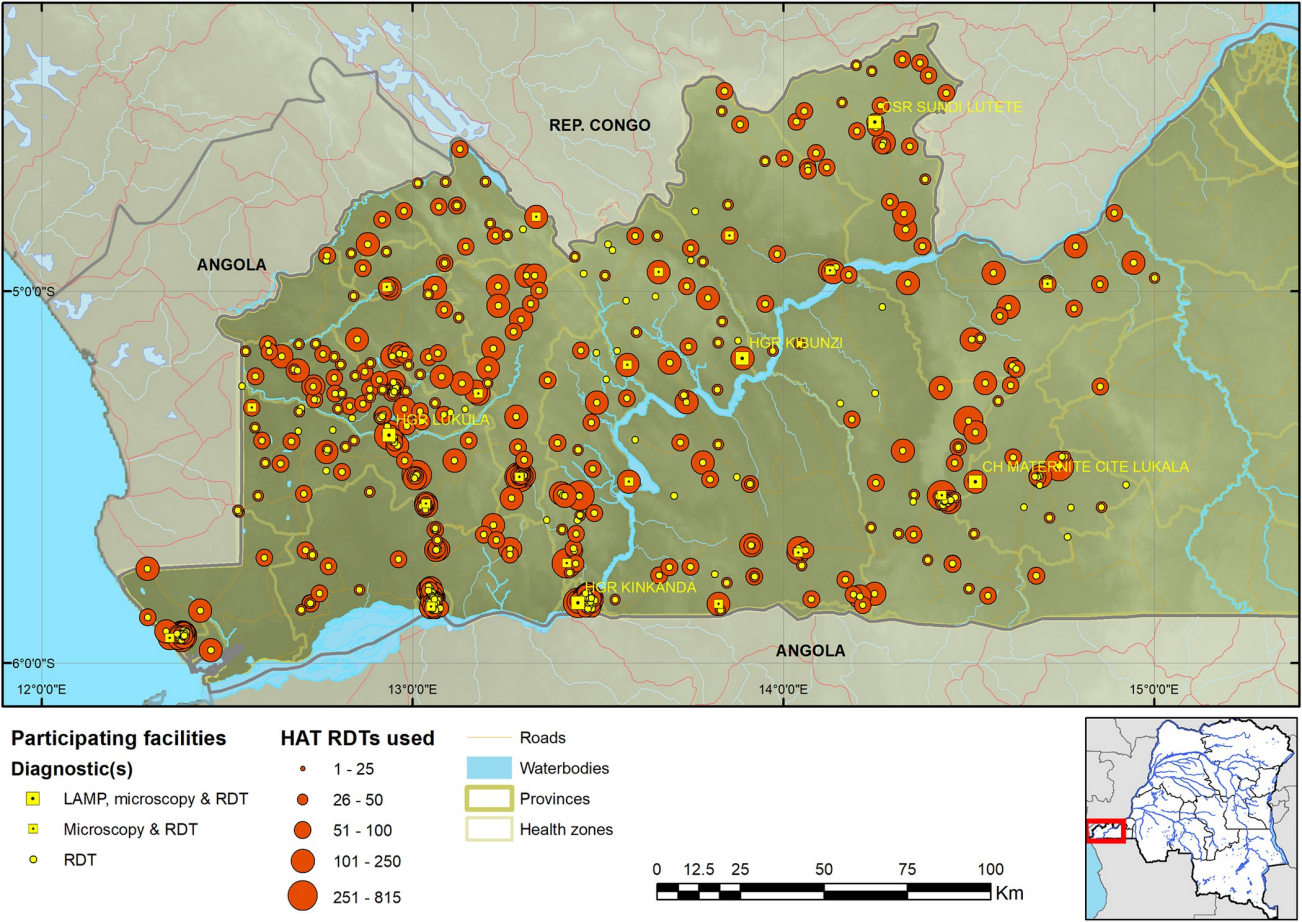
Map of Kongo Central province showing the number of RDTs performed by each health facility. The geodata layers were obtained from CC-BY License compatible sources: The CGIAR SRTM 90m datasets (https://bigdata.cgiar.org/srtm-90m-digital-elevation-database/). Further geodata were downloaded from Digital Chart of the World through Diva-GIS (https://www.diva-gis.org/gdata) and OpenStreetMaps (http://www.openstreetmap.org) and rendered by the authors using ESRI ArcGIS following the PLOS guidelines (https://journals.plos.org/plosone/s/figures) or were supplied by PNLTHA.

By the end of 2016, a total of 45,173 patients had been screened with RDTs, of whom 39,827 (88.2%) were tested at RDT facilities, 4,192 (9.3%) at microscopy facilities and 1,154 (2.6%) at LAMP facilities. Note that the number of RDT sites were 96.06% of all facilities. This was an average of 71.0 tests per RDT facility, (range 2–471, median = 60), 221 tests per HAT microscopy facility (range 67–815, median = 171) and 289 tests per LAMP facility (range 75–520, median = 280). The largest number of people were screened in the final months of 2016 (Fig 5). There was a lag in data reporting during the early stages of the programme when implementation was being initiated and reporting pathways were being streamlined. This initial lag in data reporting was sorted out by November 2015, hence the apparent spike in numbers screened during this month; thereafter, implementation, of the project improved with time, thanks to supporting activities such as supervision and monitoring (Fig 5). Utilization of HAT RDTs was relatively even across the province (Fig 6).

From the 45,173 patients tested with HAT RDTs by facilities that were still active at the end of the study, 925 (2.05%) were positive, 659 (71.2%) of them at RDT facilities. It is notable that whilst RDT facilities accounted for 88.2% of tests performed, they also accounted for 71.2% of positive tests. Out of all the RDTs performed, 1.7% were positive in RDT facilities, 4.6% in microscopy facilities and 6.5% in LAMP facilities. Therefore, the proportion of patients that were RDT +ve among all those screened (seroprevalence) was high in microscopy / LAMP sites compared to RDT sites, a difference that was statistically significant (p < 0.001). However, of the 659 patients that tested positive at RDT facilities and were referred for confirmatory testing, only 263 (39.9%) completed the referral.

### Detection of HAT cases

Out of the 296 RDT positive patients at microscopy and LAMP facilities and 263 that were successfully referred from RDT facilities, 75 were confirmed as HAT cases by microscopy during first testing, and a further 6 during a second microscopy testing after being referred with a positive LAMP test. This resulted in a total of 81 HAT cases (14.4% of the RDT positive suspects) that were detected between November 2015 and December 2016, making an average of 69.4 cases for the year. Eighteen (22.2%) of the cases were confirmed by LN, 3 (3.7%) by LED-FM, 32 (39.5%) by CTC, 22 (27.2%) by mAECT and 6 (7.4%) by CSF examination. From the 81 cases, 52 (64.2%) were in stage 1 of the disease (Table 2).

**Table 2:** HAT cases identified in Kongo Central province, DRC, by stage and type of facility that screened the patients.

		HAT stage		
		Stage 1	Stage 2	Total
Screening facility	RDT	27 (33.3%)	12 (14.8%)	39 (48.1%)
	Microscopy or LAMP *	25 (30.9%)	17* (21.0%)	42 (51.9%)
	Total	52 (64.2%)	29 (35.8%)	81 (100.0%)

* one case was not staged and was therefore managed as stage 2, in accordance with national guidelines.

**Fig 7:**
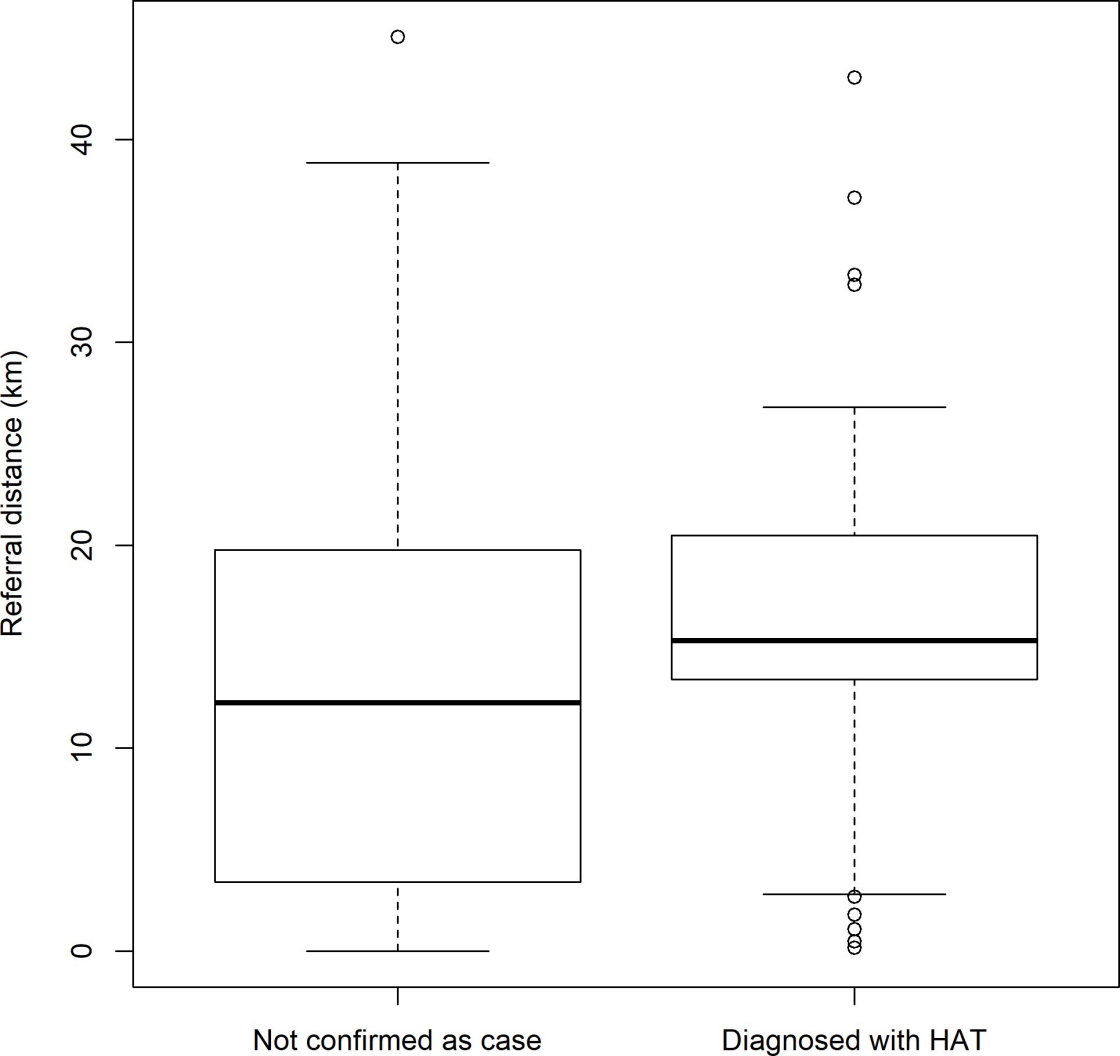
Weighted boxplot* of the referral distance from RDT facility against HAT confirmatory testing result *Boxes are weighted by the number of patients screened.

An analysis of the distance to a referral facility against whether an RDT facility screened a patient who turned out to be a HAT case shows that those RDT facilities that screened a HAT case were on average, further from a referral facility, and that those furthest from a referral facility did not screen a case (Fig 7).

The HAT detection rate among all screened people was 0.098% [95% CI: 0.070–0.134] in RDT sites and 0.786% [95% CI: 0.567–1.060] in microscopy / LAMP sites, while the HAT detection rate among RDT +ve suspects was 14.829% [95% CI: 10.762–19.709] in RDT sites (based on the RDT +ves successfully referred to microscopy / LAMP sites) and 15.789% [95% CI: 11.623–20.738] in microscopy / LAMP sites. The proportion of cases diagnosed in early stage was 71.795% [95% CI: 55.126–84.999] of cases screened in RDT sites and 59.524% [95% CI: 43.282–74.371] among the ones screened in microscopy / LAMP sites.

RDT sites, that accounted for about 95% of sites, screened about 90% of all patients. Among all patients that were RDT positive, 71% were in RDT sites. Among the RDT +ve HAT suspects referred for confirmatory testing, less than 50% (39.9%) presented at microscopy / LAMP sites, and about half (48%) of all confirmed HAT cases were first screened in RDT sites. Based on this, if a higher referral rate of RDT positive suspects had been achieved, then the proportion of diagnosed cases originating from RDT sites would have been greater than 50%.

### Reactive screening

Reactive screening was carried out in 66 villages, with a total of 30,312 people screened and 55 HAT cases identified, 50 (90.9%) of whom were in stage 1.

During the current project the 81 HAT cases identified through passive screening came from 55 villages (the origin of 1 case was unknown and 2 were from Angola), with a range of 1 to 6 cases per village. The 39 cases screened at RDT facilities came from 31 villages located at a median of between 11.5km and 63.0km from the screening site. The 42 cases screened in microscopy / LAMP sites came from 23 villages located at a median of between 18.5km and 83km from the microscopy / LAMP screening site. Among the 55 villages that reported cases passively, 20 were subjected to reactive screening, of which 8 (40%) reported 38 cases (69.1%), with 11 cases identified in one of these villages. Out of the 20 villages that reported cases passively, reactive screening took place in 45 villages linked to, or in the neighbourhood of the one that reported cases passively, that themselves didn’t report cases passively; and 6 (13.3%) of these reported 16 cases (29.1%), with 8 cases in one of them. An additional case living in one of the 55 villages that reported cases passively, not subjected to reactive screening, was detected during reactive screening in a different village subjected to reactive screening. This brought to 55 cases reported during reactive screening coming from 15 villages; 39 from 9 villages that reported cases passively and 16 cases from 6 villages that did not report cases passively, bringing to an odd ratio of villages that reported cases among the one that didn’t report cases during passive screening versus the one that reported cases of 2/3 (S2 Data).

### Comparison with former screening strategy

The strategy of screening for HAT (using CATT or RDT) implemented in this project would appear to be slightly superior compared to the period before the project. Assuming that the same number of patients were screened, this programme identified more HAT cases by passive screening (adjusted annual cases = 69.4) than those that were identified by passive screening from 2013 (53) to 2014 (30) (Table 3). Additionally, 64.2% of HAT cases identified in this programme were in stage 1, compared to 28.3% and 26.7% of cases by passive screening from 2013 to 2014 (Table 3).

**Table 3:** Comparison of the detection of HAT cases under this programme compared to the two years prior to initiation of the programme.

	2013	2014	This programme–adjusted annual numbers
Passive screening			
Numbers screened	36229	35352	39073
Cases Stage 1 (%) Stage 2 (%)	53 15 (28.3%) 38 (71.7%)	30 8 (26.7%) 22 (73.3%)	69.4 44.6 (64.2%) 24.9 (35.8%)
detection rate	0.15%	0.08%	0.18%
Active screening			
Numbers screened	51621	36626	30312
Cases Stage 1 (%) Stage 2 (%)	77 67 (87.0%) 10 (13.0%)	40 36 (90%) 4 (10%)	55 50 (90.9%) 5 (9.1%)
detection rate	0.15%	0.11%	0.18%

The HAT case numbers are adjusted because the programme was fully operational over a 14 months’ period rather than one calendar year.

The total number of HAT cases detected through passive screening during this intervention was increased by 30.94% compared to 2013, and by 131.33% compared to 2014.

While the number of patients screened for HAT during this programme would appear to be equivalent to the number screened before the study, the 40 health facilities that were active prior to the current programme screened 7,073 patients (adjusted annual screened patients = 6,063) against 36,229 and 35,352 in 2013 and 2014 respectively. During this intervention, the 40 health facilities identified 44 HAT cases (adjusted annual cases = 37.7) among all the screened patients, 29 (adjusted annual number = 24.9) of them, meaning 65.9%, in 1^st^ stage.

During the current project, active screening was conducted as reactive screening, whereby active screening is targeted at villages that reported cases passively and in their neighbourhood. We found that the detection rate during the reactive screening was higher compared to routine active screening, which targets villages that reported cases during the previous 3 to 5 years, while the proportion of cases detected in early stage was slightly higher.

## Discussion

In this paper, we have described the implementation of a programme that aims to intensify screening for HAT and increase early case finding, and thus expect to contribute to elimination of HAT in Kongo Central province, a well-circumscribed and well-delimited transboundary HAT focus in DRC, Angola and Republic of Congo. This is in the context of declining HAT incidence, which requires novel means of detecting the remaining cases in a cost-effective manner [21]. By utilising the existing health facility network that has been established by the country, with the aim of ensuring that the population is within 5 or 8km at the most from a health facility [20], the existing health facilities are used to improve population coverage.

In the DRC, Kongo Central contributes a relatively small number of cases to the national total. The results obtained in this programme can be used as an example of a strategy leading to HAT elimination, that can be implemented at a national scale. Furthermore, the Kongo Central province accounts for the majority of HAT cases in the transboundary focus of Angola, Republic of Congo and the DRC [22, 23], and therefore, driving down case numbers in this region could reduce the overall case numbers, and risk of reintroduction throughout the region. Similar passive surveillance systems that have been implemented in Angola and Republic of Congo will complement these elimination efforts. However, to envisage extending such programme to national scale, other aspects need to be considered, including the cost and feasibility.

One output that suggests that this intervention could be contributing in driving elimination is that majority of the HAT cases that were identified were in stage 1, regardless of whether they were screened at RDT or microscopy/LED facilities, meaning that they were being identified earlier in the course of infection, reducing their potential of contributing to disease transmission by acting as reservoirs. This is important in limiting disease spread (transmission) in the community. The rate at which cases present in stage 1 increased by around 40%. This suggests that people are presenting earlier in the course of infection because there is easier access to diagnostics at the local health facilities, rather than waiting until symptoms are distinctive of HAT before travelling long distances to a specialist diagnostic facility. This also means that patients’ suffering is reduced, and that they can be treated with greater safety than if they are diagnosed during stage 2. It would appear that the number of patients that were screened in the passive setting remained relatively constant when compared to previous strategies, while the number of cases identified increased. In fact, the patients screened with RDTs were systematically the clinical suspects compared to previous strategies where CATT was used as screening test. The total number of people screened in the health facilities that were active prior to the intervention was at least five times higher before the intervention compared to the number screened during the intervention. In fact, through this strategy the clinical screening was the 1^st^ screening step prior to RDT screening and therefore, the level of HAT suspicion in those tested with RDTs was higher than in previous strategies.

A core component of this strategy is the reactive screening in villages from which HAT cases were identified by passive screening or in the neighbourhoods. In regions such as Kongo Central that have a lower prevalence than other provinces of DRC, this reactive screening approach may be a good way of identifying local outbreaks. Hence, in this study, among the villages that identified cases by passive screening, 20 were followed up with reactive screening and cases identified in 8, with 11 cases in the worst affected village. Reactive screening also identified cases in 6 of 45 villages linked to or in the neighbourhood of villages where cases had been detected passively.

The detection case rate was high in this project compared to routine active screening in previous years. This may be because reactive screening as implemented in current project allows better targeting of endemic villages, but needs to be supported by a wide coverage and efficient passive screening strategy. Such passive screening would minimize the number of endemic villages that are missed by reactive screening.

The observed high rates of case detection in passive screening were in spite of the relatively poor referral rates of RDT positive suspects from RDT facilities, which was 41.2%. While it cannot be expected that this means that 58.8% of cases from RDT facilities were missed, further research would be required, to estimate the proportion of those that did not complete the referral that could have been true cases. It can however be assumed that if patients continued feeling unwell after being referred, then they would complete the referral, and that majority of patients that are not confirmed because of not completing the referral would have a self-limiting illness and will recover. However, among the 58.5% that do not present will be some that are HAT cases and did not refer due to lack or resources, time, or other reasons, or possibly died prior to completing referral. In this study, HAT detection rate among clinically suspected patients was statistically significantly higher in microscopy / LAMP sites compared to RDT sites, while there was no statistically significant difference with regard to the proportion of HAT cases among the RDT +ve suspects. The low HAT detection rate among all those screened in RDT sites may be a reflection of easy access (economic and geographic), and the fact that any patient presenting with even a few clinical signs that could be suspected of HAT are tested with the RDT (which could result in low specificity of the clinical suspicion step) compared to specialized HAT confirmatory sites. The fact that the proportion of confirmed HAT patients among all persons tested with RDT were statistically significantly higher in RDT sites than in microscopy / LAMP sites could be a reflection of an early clinical suspicion in RDT sites compared to microscopy / LAMP specialized sites. The sensitivity of the LED FM diagnostic test was low in the current intervention compared to previous findings, but did not have an impact on the performance of the whole confirmatory algorithm, as the most sensitive tests were included [6, 7]. Additional strategies, such as mobile teams that track down RDT positive suspects and perform confirmatory testing, coupled with intensified community sensitization, could improve these referral rates. Nkieri et al (2020) suggested an active follow-up implemented by Health Zone teams, using community health workers to locate suspects to be followed up [24]. The cost-effectiveness and feasibility of those strategies in relation to current strategies towards the HAT elimination goal have to be evaluated and considered.

### Limitations

This study had a number of limitations, that would need to be addressed in case such an intervention is implemented in other settings. The cost and the cost-effectiveness of this intervention to contribute to the HAT elimination objective was not evaluated. This could vary greatly depending on the accessibility in a region. The referral rate could also influence the effectiveness of such an intervention. The proportion of suspects that did not present themselves for confirmatory parasitological testing could have compromised the results. Strategies to reduce this proportion as much as possible towards 0% must be built and evaluated.

### Conclusion

The analysis presented here has demonstrated that this strategy is successful in increasing early HAT case detection, particularly in a large area (38,900km^2^) that has a relatively sparse distribution of HAT cases. Further work should be conducted to determine the costs of the strategy, including whether it could be one of the cost-effective strategies contributing to HAT elimination. In the next phases of this programme, the number of health facilities screening for HAT will be scaled back to evaluate whether a similar level of success can be achieved whilst employing a smaller number of health facilities.

## Supporting information

S1 Data Health facilities in Kongo Central province prior and during the project. (XLSX)

S2 Data Spread of HAT cases in Kongo Central province by passive and reactive screening. (XLSX)

S1 Text Health Facilities Mapping Survey Data Collection Form. (DOCX)

S1 Fig The *T. b. gambiense* human African trypanosomiasis (gHAT) diagnostic workflow implemented in Kongo Central province. Gland puncture (GP); Whole Blood (WB); Acridine Orange-Fluorescence Microscopy (AO-FM); Capillary Tube Centrifugation (CTC or mHCT); mini-Anion Exchange Centrifugation technique (mAECT); buffy-coat (bc).
(TIF)
