# Supplementary material for: Development and implementation of a strategy for intensified screening for gambiense human African trypanosomiasis in Kongo Central province, DRC
Source: PLoS Negl Trop Dis. 2020 Oct 15;14(10):e0008779. doi: 10.1371/journal.pntd.0008779 (PMC7591064; doi:10.1371/journal.pntd.0008779)
Supplement: S1 Text — (DOCX) [file pntd.0008779.s003.docx]

| **HEALTH INFRASRUCTURE MAPPING SURVEY** | | | | | | | | | | | | |  | | |  | | |  | | |  | | |  | |  |  |
| --- | --- | --- | --- | --- | --- | --- | --- | --- | --- | --- | --- | --- | --- | --- | --- | --- | --- | --- | --- | --- | --- | --- | --- | --- | --- | --- | --- | --- |
|  |  |  |  |  |  |  |  |  | | |  | | |  | | |  | | |  | | |  | | |  | |  |
| Year of survey (YYYY) | |  |  |  |  | |  |  | | |  | | |  | | |  | | |  | | |  | | |  | |  |
|  |  |  |  |  |  |  |  |  | | |  | | |  | | |  | | |  | | |  | | |  | |  |
| 1. Settlement (Village) Name (official) | | | | | |  |  | | | | | | | | | | | | | | | | | | | |  |  |
|  |  |  |  |  |  |  |  |  | | |  | | |  | | |  | | |  | | |  | | |  | |  |
| 2. Name of 1st administrative level (Province) | | | | | | |  | | | | | | | | | | | | | | | | | | | |  |  |
| 3. Name of 2nd administrative level (District) | | | | | | |  | | | | | | | | | | | | | | | | | | | |  |  |
| 4. Name of Health Zone (Zone santé) | |  |  |  |  |  |  | | | | | | | | | | | | | | | | | | | |  |  |
|  |  |  |  |  |  |  |  |  | | |  | | |  | | |  | | |  | | |  | | |  | |  |
| 5. Name of Health Infrastructure | | | | |  |  |  | | | | | | | | | | | | | | | | | | | |  |  |
|  |  |  |  |  |  |  |  |  | | |  | | |  | | |  | | |  | | |  | | |  | |  |
| 6. Geographic Coordinates of Health Infrastructure (In Decimal Degrees) | | | | | | | | | | | | | | | | | | | | | | | | |  | |  |  |
| Latitude |  |  | . |  |  |  |  |  | | |  | | |  | | |  | | |  | | |  | | |  | |  |
|  |  |  |  |  |  |  |  |  | | |  | | |  | | |  | | |  | | |  | | |  | |  |
| Longitude |  |  | . |  |  |  |  |  | | |  | | |  | | |  | | |  | | |  | | |  | |  |
|  |  |  |  |  |  |  |  |  | | |  | | |  | | |  | | |  | | |  | | |  | |  |
| 7. Estimated catchment population | | | | | |  |  |  | | |  | | |  | | |  | | |  | | | | | |  | |  |
|  |  |  |  |  |  |  |  |  | | |  | | |  | | |  | | |  | | |  | | |  | |  |
| 8. Estimated number of households in the catchment population | | |  |  |  |  |  |  | | |  | | |  | | |  | | |  | | | | | |  | |  |
|  |  |  |  |  |  |  |  |  | | |  | | |  | | |  | | |  | | |  | | |  | |  |
| 9. Year of estimated catchment population | | |  |  |  |  |  |  | | |  | | |  | | |  | | |  | | | | | |  | |  |
|  |  |  |  |  |  |  |  |  | | |  | | |  | | |  | | |  | | |  | | |  | |  |
| 10. Type of Health facility | | | |  |  | Government | | | |  | | | Private | | | | | |  | | |  | | |  | |  |  |
| Type 1 (Referal Hospital) | | | |  |  |  |  |  | | |  | | |  | | |  | | |  | | |  | | |  | |  |
|  |  |  |  |  |  |  |  |  | | |  | | |  | | |  | | |  | | |  | | |  | |  |
| Type 2 (Regional Hospital) | | | |  |  |  |  |  | | |  | | |  | | |  | | |  | | |  | | |  | |  |
|  |  |  |  |  |  |  |  |  | | |  | | |  | | |  | | |  | | |  | | |  | |  |
| Type 3 (District Hospital) | | | |  |  |  |  |  | | |  | | |  | | |  | | |  | | |  | | |  | |  |
|  |  |  |  |  |  |  |  |  | | |  | | |  | | |  | | |  | | |  | | |  | |  |
| Type 4 (Health Centre) | |  |  |  |  |  |  |  | | |  | | |  | | |  | | |  | | |  | | |  | |  |
|  |  |  |  |  |  |  |  |  | | |  | | |  | | |  | | |  | | |  | | |  | |  |
| Type 5 (Dispensary/Clinic) | | | |  |  |  |  |  | | |  | | |  | | |  | | |  | | |  | | |  | |  |
|  |  |  |  |  |  |  |  |  | | |  | | |  | | |  | | |  | | |  | | |  | |  |
| Type 6 (Health Post) | |  |  |  |  |  |  |  | | |  | | |  | | |  | | |  | | |  | | |  | |  |
|  |  |  |  |  |  |  |  |  | | |  | | |  | | |  | | |  | | |  | | |  | |  |
| Other (Specify) |  | | |  |  |  |  |  | | |  | | |  | | |  | | |  | | |  | | |  | |  |
|  |  |  |  |  |  |  |  |  | | |  | | |  | | |  | | |  | | |  | | |  | |  |
| 11. Records (History) of the last sleeping sickness case(s) | | | | | | | | | | | | | | | |  | | |  | | |  | | |  | |  |  |
| Year | 2010 | |  | 2009 | | |  | 2008 | | | | | | | | |  | | | 2007 | | | | | | | |  |
| No. of cases |  | |  |  | | |  |  | | | | | | | | |  | | |  | | | | | | | |  |
|  |  |  |  |  |  |  |  |  | | |  | | |  | | |  | | |  | | |  | | |  | |  |
|  |  |  |  |  |  |  |  |  | | |  | | |  | | |  | | |  | | |  | | |  | |  |
| 12. Capacity of the Health facility | | | | |  |  |  |  | | |  | | |  | | |  | | |  | | |  | | |  | |  |
| Human Resource capacity | | | |  |  |  |  |  | | |  | | |  | | |  | | |  | | |  | | |  | |  |
| a. Medical Doctor |  |  |  |  |  |  |  | |  | | |  | | |  | | |  | | |  | | |  | | |  |  |
|  |  |  |  |  |  |  |  |  | | |  | | |  | | |  | | |  | | |  | | |  | |  |
| b. Clinical Officer |  |  |  |  |  |  |  | |  | | |  | | |  | | |  | | |  | | |  | | |  |  |
|  |  |  |  |  |  |  |  |  | | |  | | |  | | |  | | |  | | |  | | |  | |  |
| c. Health Officer |  |  |  |  |  |  |  | |  | | |  | | |  | | |  | | |  | | |  | | |  |  |
|  |  |  |  |  |  |  |  |  | | |  | | |  | | |  | | |  | | |  | | |  | |  |
| d. Assistant Medical Officer | | | |  |  |  |  | | |  | | |  | | |  | | |  | | |  | | |  | |  |  |
|  |  |  |  |  |  |  |  |  | | |  | | |  | | |  | | |  | | |  | | |  | |  |
| e. Nurses/Nurse Assistant. | |  |  |  |  |  |  | |  | | |  | | |  | | |  | | |  | | |  | | |  |  |
|  |  |  |  |  |  |  |  |  | | |  | | |  | | |  | | |  | | |  | | |  | |  |
| f. MCH |  |  |  |  |  |  |  | |  | | |  | | |  | | |  | | |  | | |  | | |  |  |
|  |  |  |  |  |  |  |  |  | | |  | | |  | | |  | | |  | | |  | | |  | |  |
| g. Lab. Technician |  |  |  |  |  |  |  | |  | | |  | | |  | | |  | | |  | | |  | | |  |  |
|  |  |  |  |  |  |  |  |  | | |  | | |  | | |  | | |  | | |  | | |  | |  |
| h. Lab. Assistant /Attendant | |  |  |  |  |  |  | |  | | |  | | |  | | |  | | |  | | |  | | |  |  |
|  |  |  |  |  |  |  |  |  | | |  | | |  | | |  | | |  | | |  | | |  | |  |
|  |  |  |  |  |  |  |  |  | | |  | | |  | | |  | | |  | | |  | | |  | |  |
| i. Others (specify) |  |  | | | | |  | |  | | | | | |  | | |  | | |  | | |  | | |  |  |
|  |  |  |  |  |  |  |  |  | | |  | | |  | | |  | | |  | | |  | | |  | |  |
| Laboratory capacity | |  |  |  |  |  |  |  | | |  | | |  | | |  | | |  | | |  | | |  | |  |
| j. Microscope |  |  |  |  |  |  |  |  | | |  | | |  | | |  | | |  | | |  | | |  | |  |
| Electric light source | |  |  |  |  | |  |  | | |  | | |  | | |  | | |  | | |  | | |  | |  |
| Natural light source | |  |  |  |  | |  |  | | |  | | |  | | |  | | |  | | |  | | |  | |  |
|  |  |  |  |  |  |  |  |  | | |  | | |  | | |  | | |  | | |  | | |  | |  |
| k. Hematocrit centrifuge | | | |  |  |  |  | |  | | |  | | |  | | |  | | |  | | |  | | |  |  |
|  |  |  |  |  |  |  |  |  | | |  | | |  | | |  | | |  | | |  | | |  | |  |
| Reagents and Consumables available? | | | | | | |  |  | | |  | | |  | | |  | | |  | | |  | | |  | |  |
| l. Microscope Slides | | Yes |  | No |  |  |  |  | | |  | | |  | | |  | | |  | | |  | | |  | |  |
| m. Capillary tubes |  | Yes |  | No |  |  |  |  | | |  | | |  | | |  | | |  | | |  | | |  | |  |
| n. Field's/Giemsa stain | | Yes |  | No |  |  |  |  | | |  | | |  | | |  | | |  | | |  | | |  | |  |
| o. CATT tests |  | Yes |  | No |  |  |  |  | | |  | | |  | | |  | | |  | | |  | | |  | |  |
|  |  |  |  |  |  |  |  |  | | |  | | |  | | |  | | |  | | |  | | |  | |  |
| Key: |  |  |  |  |  |  |  |  | | |  | | |  | | |  | | |  | | |  | | |  | |  |
| * Community Health Worker | | | |  |  |  |  |  | | |  | | |  | | |  | | |  | | |  | | |  | |  |
| **Traditional Birth Attendant | | | |  |  |  |  |  | | |  | | |  | | |  | | |  | | |  | | |  | |  |
