# Supplementary figures and images for: Development and implementation of a strategy for intensified screening for gambiense human African trypanosomiasis in Kongo Central province, DRC
Source: PLoS Negl Trop Dis. 2020 Oct 15;14(10):e0008779. doi: 10.1371/journal.pntd.0008779 (PMC7591064; doi:10.1371/journal.pntd.0008779)

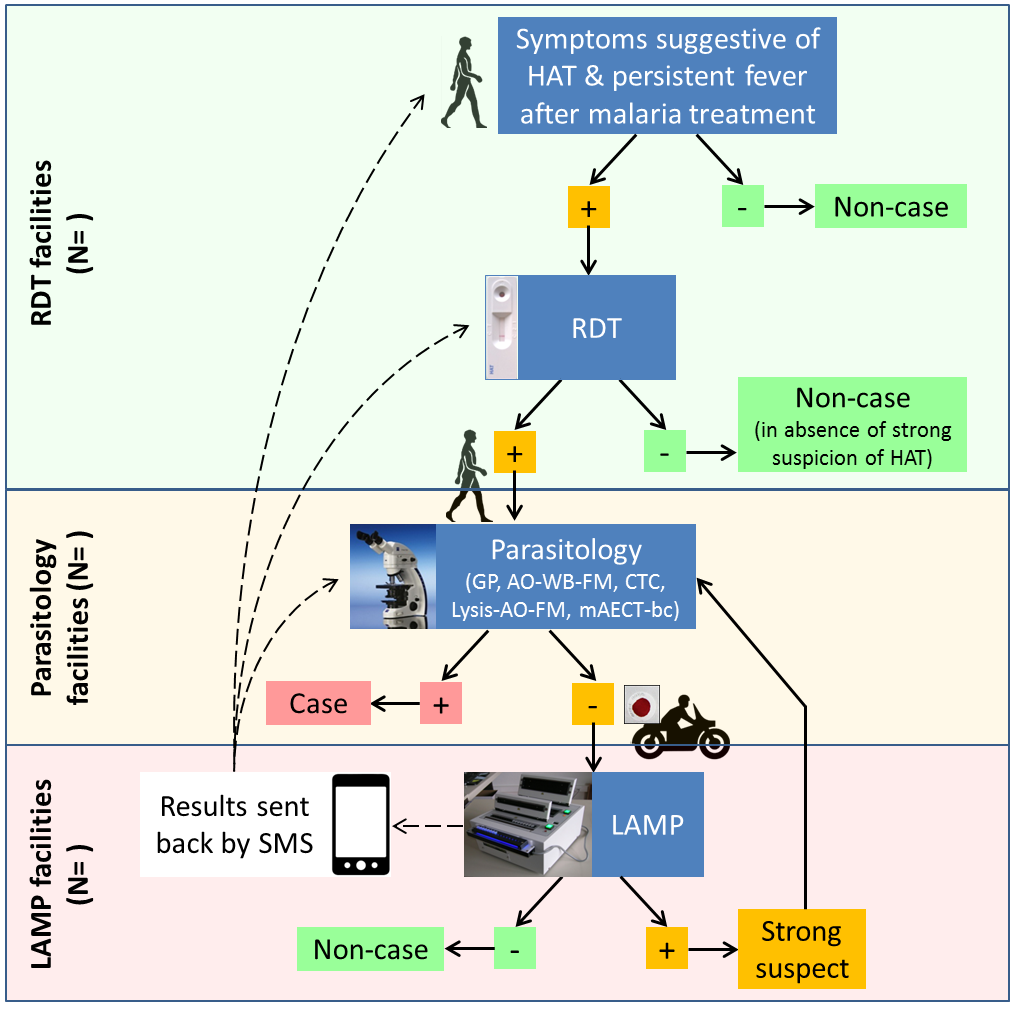

Supplement: S1 Fig — Gland puncture (GP); Whole Blood (WB); Acridine Orange-Fluorescence Microscopy (AO-FM); Capillary Tube Centrifugation (CTC or mHCT); mini-Anion Exchange Centrifugation technique (mAECT); buffy-coat (bc). (TIF) [file pntd.0008779.s004.tif]
